# Association of Myocardial Injury With Serum Procalcitonin Levels in Patients With ST-Elevation Myocardial Infarction

**DOI:** 10.1001/jamanetworkopen.2020.7030

**Published:** 2020-06-15

**Authors:** Martin Reindl, Christina Tiller, Magdalena Holzknecht, Ivan Lechner, Benjamin Henninger, Agnes Mayr, Christoph Brenner, Gert Klug, Axel Bauer, Bernhard Metzler, Sebastian J. Reinstadler

**Affiliations:** 1 Cardiology and Angiology, University Clinic of Internal Medicine III, Medical University of Innsbruck, Innsbruck, Austria; 2 University Clinic of Radiology, Medical University of Innsbruck, Innsbruck, Austria

## Abstract

**Importance:**

Myocardial tissue injury due to acute ST-elevation myocardial infarction (STEMI) initiates an inflammatory response that leads to a release of systemic inflammatory biomarkers, including C-reactive protein (CRP) and white blood cells, consequently reducing the usefulness of these routine biomarkers for identifying concomitant infections. The clinical role of procalcitonin (PCT), a promising marker of bacterial infection, to detect concomitant infection in acute STEMI is unknown, mainly because it is unclear whether myocardial injury per se induces systemic PCT release.

**Objective:**

To investigate the release of serum PCT in the acute setting of STEMI (24 and 48 hours after primary percutaneous coronary intervention) and to elucidate any associations with myocardial injury markers through a comprehensive assessment by cardiac magnetic resonance (CMR) imaging.

**Design, Setting, and Participants:**

This prospective cohort study conducted between 2016 and 2018 included 141 consecutive patients with STEMI treated with primary percutaneous coronary intervention. Concentrations of PCT, high-sensitivity CRP (hs-CRP), and high-sensitivity cardiac troponin T (hs-cTnT) and white blood cell counts were measured serially 24 and 48 hours after infarct.

**Exposures:**

Acute STEMI and primary percutaneous coronary intervention.

**Main Outcomes and Measures:**

The association of PCT and typical inflammatory marker levels with CMR-determined myocardial damage was assessed. Infarct size, extent of microvascular obstruction, and occurrence of intramyocardial hemorrhage as determined by CMR within the first week following STEMI were also evaluated.

**Results:**

In total, 141 patients with STEMI (117 men [83%]) having a median age of 56 years (interquartile range, 50-66 years) were included. The median PCT concentration was 0.07 μg/L (interquartile range, <0.06-0.11 μg/L) 24 hours after intervention and 0.07 μg/L (interquartile range, <0.06-0.09 μg/L) 48 hours after intervention. Whereas hs-CRP and hs-cTnT levels and white blood cell counts were significantly correlated with CMR markers of myocardial damage at both 24 and 48 hours after intervention, the PCT level showed no significant correlation with infarct size (at 24 hours: *r* = 0.07; *P* = .40; at 48 hours: *r* = 0.13; *P* = .12) or with microvascular obstruction (at 24 hours: *r* = −0.03; *P* = .75; at 48 hours: *r* = 0.09; *P* = .30). Furthermore, PCT levels at 24 hours (odds ratio, 1.25; 95% CI, 0.63-2.48; *P* = .52) and 48 hours (odds ratio, 1.56; 95% CI, 0.72-3.41; *P* = .26) were not significantly associated with the presence of intramyocardial hemorrhage.

**Conclusions and Relevance:**

In the acute phase after percutaneous coronary intervention for STEMI, circulating PCT levels remained unassociated with the extent of myocardial and microvascular tissue damage as visualized by CMR imaging.

## Key Points

**Question:**

Does an association exist between myocardial injury and systemic release of serum procalcitonin in acute ST-elevation myocardial infarction that is treated with primary percutaneous coronary intervention?

**Findings:**

In this cohort study of 141 patients with ST-elevation myocardial infarction, there were no significant correlations between concentration of procalcitonin 24 or 48 hours after intervention and myocardial or microvascular injury as assessed by cardiac magnetic resonance imaging.

**Meaning:**

These data highlight the clinical potential of procalcitonin to identify concomitant infection and to guide antibiotic treatments for patients with ST-elevation myocardial infarction; however, randomized trials are needed to evaluate the clinical benefit of a procalcitonin-guided strategy.

## Teaser

This cohort study evaluates whether an association exists between myocardial injury assessed with cardiac magnetic resonance imaging and the release of serum procalcitonin in patients with acute ST-elevation myocardial infarction who are treated with primary percutaneous coronary intervention.

## Introduction

Myocardial tissue injury resulting from acute ST-elevation myocardial infarction (STEMI) initiates a systemic inflammatory response that is necessary for efficient repair of the damaged myocardium.^1,2^ As part of this proinflammatory response, a plethora of cells and molecules are released into the circulation, making them potentially useful as biomarkers for risk stratification of patients with STEMI.^1,3^ Routine markers of inflammation, such as high-sensitivity C-reactive protein (hs-CRP) and white blood cell (WBC) count, have been identified as markers of infarct severity and subsequent adverse clinical outcome after STEMI.^4,5,6^

However, in the setting of supposed concomitant infections that complicate STEMI, the interpretation of these inflammatory biomarkers are challenging, frequently leading to the misuse of antibiotics in daily clinical practice.^7,8^ Procalcitonin (PCT) has emerged as a useful clinical biomarker to improve diagnosis of bacterial infection and to guide antibiotic therapy.^9,10^ For patients with acute heart failure, PCT has been shown to optimize the detection of superimposed infections, and a PCT-guided approach has been suggested previously for the use of antibiotics for patients with acute heart failure.^11,12^ In acute STEMI, however, the significance of PCT for the detection of concomitant infection is less clear. In a previous analysis of patients with myocardial infarction, including those with or without STEMI, PCT showed higher accuracy for the diagnosis of coexisting infections than other markers, such as CRP and WBC count.^8^ In line with this finding, there is evidence that myocardial infarction per se does not induce a significant systemic PCT release.^13,14^ By contrast, other earlier studies have shown PCT as an indicator of myocardial damage within the context of infarction.^15,16^ Given these controversial data, there is a need for dedicated studies that explicitly evaluate the association between PCT concentrations and infarct severity to improve our understanding of the clinical role of PCT in acute myocardial infarction.

Cardiac magnetic resonance (CMR) imaging enables precise evaluation and quantification of myocardial and microvascular tissue injury and, therefore, represents the current in vivo criterion standard for the assessment of infarct severity after STEMI.^17^ However, data on the association of PCT with CMR-determined myocardial injury after STEMI are entirely lacking. Therefore, we investigated the association of serum PCT concentration with infarct severity as assessed by comprehensive CMR imaging among patients with acute STEMI who were treated with primary percutaneous coronary intervention.

## Methods

### Study Design, Participants, and Biomarker Analyses

In this prospective cohort study, 141 consecutive patients with STEMI who were enrolled in the Magnetic Resonance Imaging In Acute STEMI (MARINA-STEMI) trial were included. The MARINA-STEMI trial is a large prospective cohort study aiming to evaluate the clinical role and prognostic validity of CMR parameters of myocardial tissue injury for patients with STEMI. The present analysis represents a predefined biomarker substudy and included patients enrolled between 2016 and 2018. In total, 150 STEMI patients initially met the inclusion criteria. Of these 150 patients, 4 developed hemodynamic deterioration after inclusion and 5 showed insufficient CMR data (4 with reduced image quality or artifacts, and 1 with premature termination of the CMR scan by the patient). This resulted in a final cohort of 141 stable patients with STEMI having appropriate CMR data. This study followed the Strengthening the Reporting of Observational Studies in Epidemiology (STROBE) reporting guideline for cohort studies. The research ethics committee at the Medical University of Innsbruck approved the study, and the investigation was conducted following the Declaration of Helsinki. Before inclusion, all participants gave written informed consent. No participant received compensation or was offered any incentive for participating in this study.

Inclusion criteria included a first-ever STEMI defined as having symptoms of ischemia and ST-segment elevation of at least 0.1 mV in 2 contiguous extremity leads or at least 0.2 mV in 2 contiguous precordial leads.^18,19^ Additional inclusion criteria included revascularization by primary percutaneous coronary intervention within 24 hours after onset of symptoms, being older than 18 years of age, having an estimated glomerular filtration rate greater than 30 mL/min/1.73 m^2^, and having Killip classification lower than III at time of CMR. Exclusion criteria were defined as any history of previous myocardial infarction or coronary intervention and contraindications to CMR (ie, pacemaker, claustrophobia, orbital foreign body, cerebral aneurysm clip, and known or suggested contrast agent allergy to gadolinium). All patients with clinical evidence of infection were also excluded (ie, pulmonary infiltrates observed on chest radiographic image, fever, and dysuria).

A detailed medical history and comprehensive physical examination (including auscultation of the heart and lungs and measurement of temperature) were taken on admission, as well as once daily during the hospital stay. Chest radiography was conducted for all patients within 24 hours after the intervention. Biochemical measurements of inflammatory markers high-sensitivity cardiac troponin T (hs-cTnT) and creatinine were performed serially on day 1 and day 2 after primary percutaneous coronary intervention. Analyses of PCT and hs-CRP were conducted using the Cobas 8000 Modular Analyzer (Roche Diagnostics). The functional sensitivity of the PCT assay was 0.06 μg/L. Based on previous recommendations for the use of antibiotics in outpatient and emergency department settings,^20^ a PCT threshold of 0.25 μg/L defined an increased PCT concentration. Concentrations of hs-cTnT were determined using a validated enzyme immunoassay (E170; Roche Diagnostics).^21^

### Cardiac Magnetic Resonance Imaging

All CMR examinations were conducted using a 1.5-T Magnetom Avanto scanner (Siemens) 4 days following infarction (interquartile range [IQR], 3-5 days). The standardized imaging protocol and image postprocessing used by members of our research group was applied.^22^ In brief, left ventricular dimensions and function were determined on short-axis cine images (10-12 slices; electrocardiography-triggered TrueFISP bright-blood sequences). The accompanying standard software (Argus; Siemens) was used for postprocessing. For myocardial damage evaluation, late gadolinium enhancement images were acquired 15 minutes after administration of a bolus of contrast agent (Gadovist, 0.2 mmol/kg; Bayer) using an electrocardiography-triggered phase-sensitive inversion recovery sequence.^23^ Consistent with previous reports, hyperenhancement was defined by a threshold of 5 SDs higher than the signal intensity of remote myocardium in the opposite left ventricular myocardial segment.^24,25^ Infarct size (IS) was presented as a percentage of the entire left ventricular myocardial mass. Microvascular obstruction (MVO) was defined as an area of “hypoenhancement” within the infarcted territory.^26^ Both the presence and extent (percentage of left ventricular myocardial mass) of MVO was ascertained. The presence of intramyocardial hemorrhage (IMH) was evaluated using T2* mapping as described in detail previously.^27^ The T2* data were not available in 6 patients owing to poor image quality. Experienced observers (C.T. and A.M.), blinded to all clinical data, conducted the CMR measurements and analyses.

### Statistical Analysis

**Figure 1.**
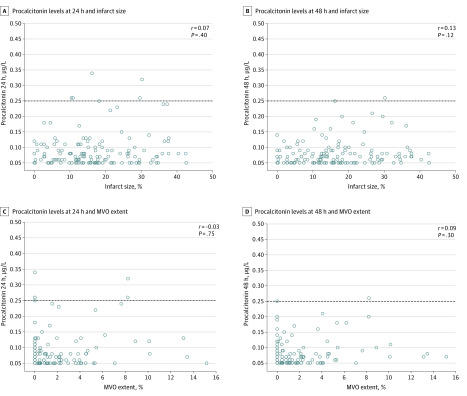
Correlation of Procalcitonin Concentrations With Myocardial Injury Scatterplots display the correlation between procalcitonin levels (at 24 and at 48 hours after primary percutaneous coronary intervention) with infarct size (A and B) and with the extent of microvascular obstruction (MVO) (C and D). Dashed line at 0.25 μg/L indicates the threshold for clinically relevant procalcitonin increase.

The software SPSS, version 25.0 (IBM), and MedCalc Statistical Software, version 15.8, were used for statistical analyses. Continuous variables are presented as medians with the corresponding IQRs, and categorical variables are expressed as a number with the corresponding percentage. Correlations between continuous variables were assessed using the Spearman ρ test. The correlations of PCT with IS and of PCT with MVO are illustrated using scatter plots (Figure 1). The differences in continuous variables between 2 groups were evaluated using the Mann-Whitney test. Differences in categorical variables were evaluated using the χ^2^ test. Associations of continuous variables (biomarkers) with a binary dependent variable (IMH) were assessed by logistic regression analysis. All tests were 2-sided, and *P* < .05 was defined as significant for all statistical tests.

## Results

### Baseline Characteristics

In the present analysis, 141 patients with STEMI (median age, 56 years [IQR, 50-66 years]; 117 men [83%] and 24 women [17%]) with a total ischemia time of 183 minutes (IQR, 115-285 minutes) were included. The baseline demographic and clinical characteristics of the patients, including cardiovascular risk factors, angiographic findings, biochemical markers, and CMR parameters, are provided in Table 1.

**Table 1.** Demographic and Clinical Characteristics of Patients

Characteristic	Patients, No. (%)			*P* value
	Total population	IS <15%	IS ≥15%	
No. (%)	141 (100)	70 (50)	71 (50)	
Age, median (IQR), y	56 (50-66)	56 (49-62)	57 (51-67)	.35
Female	24 (17)	9 (13)	15 (21)	.19
Hypertension	66 (47)	30 (43)	36 (51)	.35
Current smoker	76 (54)	40 (57)	36 (51)	.44
Hyperlipidemia	79 (56)	39 (56)	40 (56)	.94
Diabetes	17 (12)	8 (11)	9 (13)	.82
Total ischemia time, median (IQR), min	183 (115-285)	181 (108-272)	184 (120-293)	.52
Culprit lesion				
RCA	54 (38)	36 (51)	18 (25)	.01
LAD	66 (47)	24 (34)	42 (59)	
LCX	19 (14)	9 (13)	10 (14)	
RI	2 (1)	1 (1)	1 (1)	
No. of diseased vessels				
1	88 (62)	45 (64)	43 (61)	.16
2	38 (27)	21 (30)	17 (24)	
3	15 (11)	4 (6)	11 (16)	
Creatinine, median (IQR), mg/dL				
24 h	0.94 (0.82-1.10)	0.92 (0.82-1.08)	0.95 (0.81-1.11)	.57
48 h	0.98 (0.87-1.11)	0.97 (0.88-1.10)	0.99 (0.85-1.12)	.71
hs-cTnT, median (IQR), ng/L				
24 h	3376 (1614-5357)	1721 (824-3210)	5242 (3730-7162)	<.001
48 h	2596 (1419-4361)	1490 (831-2549)	3788 (2678-5677)	<.001
hs-CRP, median (IQR), mg/dL				
24 h	1.63 (0.90-2.74)	1.27 (0.75-1.88)	2.04 (1.29-3.51)	<.001
48 h	2.88 (1.52-4.43)	1.88 (1.01-3.64)	3.79 (2.33-6.53)	<.001
WBC count, median (IQR), cells/μL				
24 h	10 800 (8600-12 800)	9600 (7900-12 300)	11 500 (10 000-13 700)	<.001
48 h	8800 (7700-10 400)	8300 (7200-9700)	9500 (8200-10 700)	<.001
Procalcitonin, median (IQR), μg/L				
24 h	0.07 (<0.06-0.11)	0.07 (<0.06-0.11)	0.07 (<0.06-0.11)	.86
48 h	0.07 (<0.06-0.09)	0.07 (<0.06-0.09)	0.07 (<0.06-0.09)	.31
CMR imaging parameters				
LVEF, %	49 (42-56)	54 (47-58)	45 (38-52)	<.001
LVEDV, mL	151 (130-176)	147 (130-171)	152 (128-184)	.36
LVESV, mL	77 (58-98)	68 (57-82)	85 (64-106)	.001
IS, % of LVMM	15 (8-25)	8 (4-12)	24 (18-32)	<.001
MVO				
No. (%)	81 (57)	23 (33)	58 (82)	<.001
% Of LVMM	0.4 (0.0-2.3)	0.0 (0.0-0.4)	2.1 (0.5-4.5)	<.001

Abbreviations: CMR, cardiac magnetic resonance; hs-CRP, high-sensitivity C-reactive protein; hs-cTnT, high-sensitivity cardiac troponin T; IQR, interquartile range; IS, infarct size; LAD, left anterior descending artery; LCX, left circumflex artery; LVEDV, left ventricular end diastolic volume; LVEF, left ventricular ejection fraction; LVESV, left ventricular end-systolic volume; LVMM, left ventricular myocardial mass; MVO, microvascular obstruction; RCA, right coronary artery; RI, ramus intermedius; WBC, white blood cell.

SI conversion factors: To convert serum creatinine to μmol/L, multiply by 76.25; hs-CRP to mg/L, by 10; and WBC to cells ×10^9^/L, by 0.001.

### Inflammatory Markers

Median concentrations of PCT were 0.07 μg/L (IQR, <0.06 to 0.11 μg/L) 24 hours after intervention and 0.07 μg/L (IQR, <0.06 to 0.09 μg/L) at 48 hours. Of 141 patients, at 24 hours, 41 patients (29%) showed a PCT concentration below the functional sensitivity cutoff of 0.06 μg/L. At 48 hours, 47 patients (33%) had PCT concentrations less than 0.06 μg/L. At 24 hours, 42 patients (30%) had a PCT concentration of 0.10 μg/L or higher, and at 48 hours, 30 patients (21%) had a PCT concentration of 0.10 μg/L or higher. A PCT concentration of 0.25 μg/L or higher was detected in 7 patients (5%) at 24 hours and in 3 patients (2%) at 48 hours. The median hs-CRP level increased from 1.63 mg/dL (IQR, 0.90-2.74 mg/dL) at 24 hours to 2.88 mg/dL (IQR, 1.52-4.43 mg/dL) at 48 hours (to convert to mg/L, multiply by 10), whereas the median WBC count decreased from 10 800 cells/μL (IQR, 8600-12 800 cells/μL) to 8800 cells/μL (7700-10 400 cells/μL) (to convert to cells ×10^9^/L, multiply by 0.001).

### Inflammatory Markers and Myocardial Damage

The correlations of inflammatory markers and hs-cTnT levels with IS and MVO extent are given in Table 2. Whereas hs-CRP and hs-cTnT levels and WBC counts were significantly correlated with both markers of myocardial damage 24 and 48 hours after intervention, PCT showed no significant correlation with IS (at 24 hours: *r* = 0.07, *P* = .40; at 48 hours: *r* = 0.13, *P* = .12) or with MVO (at 24 hours: *r* = −0.03, *P* = .75; at 48 hours: *r* = 0.09, *P* = .30). The correlations of PCT levels with IS and with MVO are further illustrated in Figure 1 (scatterplots).

**Table 2.** Correlations of Inflammatory Biomarkers With Myocardial Injury Markers 24 and 48 Hours After Primary Percutaneous Coronary Intervention

Biomarker	% of left ventricular myocardial mass			
	Infarct size		Microvascular obstruction	
	*r* Value	*P* value	*r* Value	*P* value
hs-cTnT level				
24 h	0.80	<.001	0.60	<.001
48 h	0.75	<.001	0.55	<.001
hs-CRP level				
24 h	0.42	<.001	0.38	<.001
48 h	0.50	<.001	0.54	<.001
WBC count				
24 h	0.33	<.001	0.35	<.001
48 h	0.32	<.001	0.35	<.001
Procalcitonin level				
24 h	0.07	.40	−0.03	.75
48 h	0.13	.12	0.09	.30

Abbreviations: hs-CRP, high-sensitivity C-reactive protein; hs-cTnT, high-sensitivity cardiac troponin T; WBC, white blood cell.

The median IS of the study cohort was 15% (IQR, 8%-25%). Differences in baseline characteristics between patients with IS less than 15% and patients with IS 15% or higher are presented in Table 1. Compared with patients with a small IS (<15%), those with large IS (≥15%) showed significantly higher concentrations of hs-CRP (median concentration: 1.27 mg/dL [IQR, 0.75-1.88 mg/dL] for IS <15% vs 2.04 mg/dL [IQR, 1.29-3.51 mg/dL] for IS ≥15% at 24 hours and 1.88 mg/dL [IQR, 1.01-3.64 mg/dL] for IS <15% vs 3.79 mg/dL [IQR, 2.33-6.53 mg/dL] for IS ≥15% at 48 hours) and hs-cTnT (median concentration: 1721 ng/L [IQR, 824-3210 ng/L] for IS <15% vs 5242 ng/L [IQR, 3730-7162 ng/L] for IS ≥15% at 24 hours and 1490 ng/L [IQR, 831-2549 ng/L] for IS <15% vs 3788 ng/L [IQR, 2678-5677 ng/L] for IS ≥15% at 48 hours) and of WBC counts (median concentration: 9600 cell/μL [IQR, 7900-12 300 cell/μL] for IS <15% vs 11 500 cell/μL [IQR, 10 000-13 700 cell/μL] for IS ≥15% at 24 hours and 8300 cell/μL [IQR, 7200-9700 cell/μL] for IS <15% vs 9500 cell/μL [IQR, 8200-10 700 cell/μL] for IS ≥15% at 48 hours) at both time points (*P* < .001 for all), whereas PCT concentrations were not significantly associated with IS at 24 hours (0.07 μg/L [IQR, <0.06-0.11 μg/L] for IS <15% vs 0.07 μg/L [IQR, <0.06-0.11 μg/L] for IS ≥15%; *P* = .86) or at 48 hours (0.07 μg/L [IQR, <0.06-0.09 μg/L] for IS <15% vs 0.07 μg/L [IQR, <0.06-0.09 μg/L] for IS ≥15%; *P* = .31).

With the use of CMR imaging, 81 patients (57%) displayed MVO, and the median MVO was 0.4% (IQR, 0.0%-2.3%). For patients with MVO 0.4% or higher, significantly higher concentrations of hs-CRP (median concentration: 1.99 mg/dL [IQR, 1.28-3.56 mg/dL] at 24 hours; 4.13 mg/dL [IQR, 2.50-6.61 mg/dL] at 48 hours [*P* < .001]) and hs-cTnT (median concentration: 4908 ng/L [IQR, 3357-6573 ng/L] at 24 hours; 3727 ng/L [IQR, 2456-5488 ng/L] at 48 hours [*P* < .001]) and higher WBC counts (median count: 11 800 cells/μL [IQR 9600-13 700 cells/μL] at 24 hours [*P* < .001]; 9400 cells/μL [IQR, 8100-10 800 cells/μL] at 28 hours [*P* = .001]) were detected. However, PCT concentrations were not significantly different for patients with MVO 0.4% or higher (median concentration: 0.07 μg/L [IQR, <0.06 to 0.10 μg/L] at 24 hours; 0.07 μg/L [IQR, <0.06 to 0.09 μg/L] at 48 hours) compared with patients with MVO lower than 0.4% (median concentration: 0.08 μg/L [IQR, <0.06 to 0.11 μg/L] at 24 hours [*P* = .39]; 0.07 μg/L [IQR, <0.06 to 0.09 μg/L] at 48 hours [*P* = .88]). The concentrations of the biomarkers based on MVO extent are given in the eTable in the Supplement.

**Figure 2.**
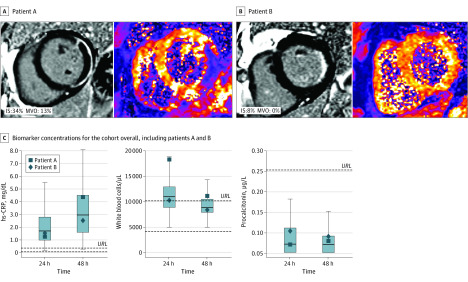
Inflammatory Biomarkers in Acute ST-Segment Elevation Myocardial Infarction (STEMI) Systemic release of inflammatory biomarkers illustrated with 2 representative patients with lateral wall STEMI. A and B, Images of late gadolinium-enhanced cardiovascular magnetic resonance imaging (left panels) and T2*-mapping (right panels) of each patient. C, Boxplots illustrating biomarker concentrations for the overall cohort 24 and 48 hours after primary percutaneous coronary intervention. Gray boxes represent interquartile ranges; horizontal lines within these boxes, median concentrations; ends of whiskers, minimum and maximum concentrations; dark blue boxes, biomarker concentrations for patient A; dark blue diamonds, biomarker concentrations for patient B; dashed lines, reference range for each biomarker; URL, upper reference limit. Patient A with large (34%) infarct size (IS) and large (13%) microvascular obstruction (MVO) (panel A, left) and presence of intramyocardial hemorrhage (IMH) (panel A, right) shows a marked increase in levels of high-sensitivity C-reactive protein (hs-CRP; to convert mg/L to nmol/L, multiply by 9.524) and white blood cell count (to convert to cells ×10^9^/L, multiply by 0.001), whereas the procalcitonin level remains low. Patient B has a small IS (8%) without MVO (panel B, left) or IMH (panel B, right). This patient shows only slightly increased hs-CRP levels and a normal WBC count with no significant elevation in procalcitonin level.

Of 135 patients with T2* data, 44 patients (33%) displayed IMH. Compared with patients without IMH, those with IMH showed significantly higher concentrations of hs-CRP (at 24 hours: odds ratio [OR], 2.20 [95% CI, 1.39-3.48]; *P* = .001; at 48 hours: OR, 2.10 [95% CI, 1.33-3.32]; *P* = .002), WBC count (at 24 hours: OR, 5.76 [95% CI, 1.47-22.56]; at 48 hours: OR, 8.99 [95% CI, 1.85-43.61]; *P* = .01 for both) and hs-cTnT levels (at 24 hours: OR, 4.86 [95% CI, 2.49-9.52]; at 48 hours: OR, 3.36 [95% CI, 1.99-6.63]; *P* < .001 for both). By contrast, PCT did not differ significantly between patients with or without IMH (at 24 hours: OR, 1.25 [95% CI, 0.63-2.48]; *P* = .52; at 48 hours: OR, 1.56 [95% CI, 0.72-3.41]; *P* = .26). The associations of inflammatory biomarkers with IMH as determined by logistic regression analyses are presented in Table 3. Figure 2 shows 2 representative patients illustrating the association between the systemic release of inflammatory biomarkers and myocardial injury.

**Table 3.** Univariable Logistic Regression Analysis of Intramyocardial Hemorrhage 24 and 48 Hours After Primary Percutaneous Coronary Intervention^a^

Biomarker	Odds ratio (95% CI)	*P* value
hs-cTnT level		
24 h	4.86 (2.49-9.52)	<.001
48 h	3.36 (1.99-6.63)	<.001
hs-CRP level		
24 h	2.20 (1.39-3.48)	.001
48 h	2.10 (1.33-3.32)	.002
WBC count		
24 h	5.76 (1.47-22.56)	.01
48 h	8.99 (1.85-43.61)	.01
Procalcitonin level		
24 h	1.25 (0.63-2.48)	.52
48 h	1.56 (0.72-3.41)	.26

Abbreviations: hs-CRP, high-sensitivity C-reactive protein; hs-cTnT, high-sensitivity cardiac troponin T; WBC, white blood cell.

^a^ All variables were log-transformed for this analysis.

## Discussion

The present study investigated the association of serum PCT concentration with the extent of myocardial and microvascular injury that were comprehensively evaluated by CMR among patients with STEMI treated with primary percutaneous coronary intervention. The key findings of the present investigation were that (1) whereas hs-CRP levels and WBC counts serially measured after STEMI showed significant associations with IS and with MVO, (2) PCT concentrations showed no significant association with the extent of myocardial or microvascular damage; (3) indeed, in contrast to hs-CRP and WBCs, no detectable release of PCT into the circulation was observed in the acute setting of STEMI. These data confirm and extend the knowledge base of previous studies because here we highlight that in stable patients with STEMI, circulating PCT concentrations were unassociated with myocardial tissue injury as directly depicted by multiparametric CMR imaging. Accordingly, there is a potential for PCT to be used as a necrosis-independent clinical biomarker to detect concomitant infection and guide antibiotic therapies for patients with STEMI. However, future studies must be conducted to confirm this hypothesis by including patients with STEMI who also manifest infection.

Inflammatory markers are of crucial importance for the detection of infectious disease as well as for accurate therapeutic decision-making in everyday clinical practice.^28^ However, the commonly used inflammatory biomarkers, including hs-CRP and WBCs, are not only released in the setting of infections but also represent nonspecific markers of inflammation in general.^29^ Hence, in clinical scenarios of aseptic inflammation, the usefulness of inflammatory biomarkers to identify concomitant infectious diseases is limited. Acute myocardial infarction represents a clinical scenario of systemic inflammatory activation in response to myocardial injury.^1,2^ Indeed, several previous studies have shown a significant increase in circulating levels of hs-CRP and WBCs in acute infarctions.^1,6^ Furthermore, hs-CRP levels and WBC counts have been correlated with the extent of myocardial and microvascular damage in patients with STEMI,^4,6^ which is consistent with the results of the present analyses.

The precursor molecule of the peptide hormone calcitonin, PCT, has been revealed as a valuable biomarker for the detection of bacterial infections and for the guidance of antibiotic treatments in different patient populations.^9^ The BACH trial by Maisel et al,^11^ which included more than 1600 patients who presented to the emergency department with dyspnea, showed that in the subgroup of patients with acute heart failure, the use of PCT levels was associated with an increase in accuracy for diagnosing coexisting pneumonia and that a PCT-guided strategy may improve antibiotic therapy decision-making. Based on those data, the latest European guidelines recommend PCT assessment for differential diagnosis and the use of antibiotics in acute heart failure.^12^ However, for patients with acute myocardial infarction, in which ischemic myocardial injury and the associated inflammatory response might influence PCT release and thus bias the validity of PCT to diagnose coexisting infections, the role of PCT remained unclear. Existing literature on this issue is scarce and inconclusive.^8^ In a study by Vitkon-Barkay et al,^8^ 230 patients with acute myocardial infarction were evaluated by infectious diseases specialists blinded to the PCT results. Their findings indicated that the use of PCT level outperformed the use of the CRP level or WBC count in detecting coexisting infections. Explaining the superior discriminative power of PCT levels over CRP levels and WBC counts, earlier data have suggested that uncomplicated myocardial infarction does not lead to an increase in PCT levels.^13,14,30^ By contrast, other previous reports state that myocardial infarction per se is associated with a significant release of PCT.^31,32^ Moreover, PCT was proposed as a sensitive marker of myocardial damage as well as being associated with left ventricular remodeling and worse clinical outcome following infarction.^15,16,32^ Taken together, these limited and controversial previous data cannot clarify the clinical significance of PCT in the setting of acute myocardial infarction. In particular, the fundamental question in this context, namely, whether cardiac damage affects circulating PCT levels, could not be answered by previous studies owing to a lack of suitable methods for adequate assessment and quantification of myocardial injury. The present investigation used comprehensive CMR imaging, which is the in vivo method of choice for the evaluation of myocardial and microvascular injury in acute myocardial infarction,^33^ to find that circulating PCT concentrations were not associated with the damaged myocardium in acute STEMI. Indeed, neither IS nor extent of MVO showed significant associations with serum PCT levels. In addition, the vast majority (95%) of the included patients with STEMI patients had a PCT concentration lower than 0.25 μg/L, emphasizing that myocardial tissue injury due to acute STEMI by itself is not associated with clinically relevant levels of PCT release. These crucial data suggest that in acute STEMI treated with primary percutaneous coronary intervention, PCT does not act as an unspecific biomarker (such as hs-CRP or WBCs) that is involved in the general inflammatory response reaction following infarction. Consequently, PCT provides great clinical potential to identify coexisting infections and facilitate antibiotic treatment decisions for patients with acute STEMI. This hypothesis warrants prospective evaluation in appropriately designed clinical trials.

### Limitations

We included stable patients with STEMI having Killip class I or II in the present analysis. Accordingly, our findings do not apply to hemodynamically unstable patients with STEMI although they represent only a minority of all patients with STEMI.^34^ A previous study revealed that in contrast to patients with stable infarction, patients with cardiogenic shock show a clinically relevant increase in PCT concentration despite the absence of infection,^14^ highlighting that in the clinical context of PCT level interpretation, stable and critically ill cardiac patients need to be differentiated. The mechanisms explaining the systemic PCT release in patients with cardiogenic shock are incompletely understood; however, bacterial endotoxin exposure due to bowel wall congestion, ischemia, or both has been suggested as a possible underlying pathophysiological link.^35^ This association between PCT and hemodynamic instability may also provide a plausible explanation for the significant increase in PCT levels detected by previous analyses, including unselected patients experiencing acute coronary syndrome.^31,32^ Indeed, the study by Kafkas et al^32^ showed a significant association of PCT with Killip class, however, not with enzymatic infarct severity.

On the basis of prior studies in which a significant PCT level increase was detected in the first 24 to 48 hours after infarction,^31,32^ PCT measurements in the present study were performed in that same time range. We, therefore, cannot provide information concerning PCT levels and dynamics in the subacute or chronic phase that follows STEMI.

Finally, because the present study aimed to assess the association between PCT and myocardial damage per se, patients with STEMI and infection were excluded. Hence, the promising clinical role of PCT for patients with STEMI and infection and the usefulness of PCT-guided therapeutic approaches remain to be clarified by future studies.

## Conclusions

For patients with STEMI treated with percutaneous coronary intervention, hs-CRP levels and WBC counts but not PCT concentrations were associated with the degree of myocardial or microvascular injury as determined by comprehensive CMR imaging. Furthermore, in contrast to hs-CRP level and WBC count, no clinically relevant increase in serum PCT level was detected in the acute setting of STEMI. These data indicated that PCT remained unassociated with the processes of myocardial tissue injury in acute STEMI, suggesting that PCT may be a promising biomarker in detecting, or excluding, concomitant bacterial infection, which can help to guide therapeutic antibiotic decision-making for these patients. Nonetheless, further validation in randomized clinical trials is required.

## Notes

Supplement. **eTable.** Biomarker Concentrations According to MVO Extent
