# Supplementary material for: Association of Myocardial Injury With Serum Procalcitonin Levels in Patients With ST-Elevation Myocardial Infarction
Source: JAMA Netw Open. 2020 Jun 15;3(6):e207030. doi: 10.1001/jamanetworkopen.2020.7030 (PMC7296390; doi:10.1001/jamanetworkopen.2020.7030)
Supplement: Supplement. — eTable. Biomarker Concentrations According to MVO Extent [file jamanetwopen-3-e207030-s001.pdf]

Supplementary Online Content

Reindl M, Tiller C, Holzknecht M, et al. Association of myocardial injury with serum procalcitonin levels in patients with ST-elevation myocardial infarction. *JAMA Netw Open*. 2020;3(6):e207030. doi:10.1001/jamanetworkopen.2020.7030

eTable. Biomarker Concentrations According to MVO Extent

This supplementary material has been provided by the authors to give readers additional information about their work.

© 2020 Reindl M et al. *JAMA Network Open*.

eTable. Biomarker Concentrations According to MVO Extent

	MVO <0.4% (n=70, 50%)	MVO ≥0.4% (n=71, 50%)	*P* value
**Hs-cTnT 24h, ng/l**	1721 [976-3379]	4908 [3357-6573]	**<.001**
**Hs-cTnT 48h, ng/l**	1530 [851-3025]	3727 [2456-5488]	**<.001**
**Hs-CRP 24h, mg/l**	13.1 [7.4-19.7]	19.9 [12.8-35.6]	**<.001**
**Hs-CRP 48h, mg/l**	18.2 [9.9-31.5]	41.3 [25.0-66.1]	**<.001**
**WBCc 24h, 10^9/l**	9.8 [7.8-11.7]	11.8 [9.6-13.7]	**<.001**
**WBCc 48h, 10^9/l**	8.3 [6.9-9.7]	9.4 [8.1-10.8]	**.001**
**Procalcitonin 24h, µg/l**	0.08 [<0.06-0.11]	0.07 [<0.06-0.10]	.39
**Procalcitonin 48h, µg/l**	0.07 [<0.06-0.09]	0.07 [<0.06-0.09]	.88

Hs-CRP=High-sensitivity C-reactive protein; Hs-cTnT=High-sensitivity cardiac Troponin T; MVO=Microvascular obstruction; WBCc=White blood cell count.

© 2020 Reindl M et al. *JAMA Network Open*.
